# Selection and validation of emotional videos: Dataset of professional and amateur videos that elicit basic emotions

**DOI:** 10.1016/j.dib.2020.106662

**Published:** 2020-12-20

**Authors:** HongYi Chen, Kai Ling Chin, Chrystalle B.Y. Tan

**Affiliations:** a Faculty of Medicine and Health Sciences, Universiti Malaysia Sabah, Malaysia; b Department of Medicine Nursing, TongRen Polytechnic College, China

**Keywords:** Cross-cultural, Emotional videos, Emotion elicitation, Emotion recognition, Emotional intensity

## Abstract

This article describes the process of selecting a collection of professional and amateur videos that elicit five basic emotions (i.e., happiness, fear, disgust, anger, and sadness) and validating these videos in three groups of participants (i.e., Chinese from China, Chinese from Malaysia, and Bumiputera from Malaysia). In the video selection phase, professional videos, which were Western movie trailers, were selected from IMDb (Internet Movie Database) and amateur videos were selected from YouTube. The researchers selected videos that display five basic emotions, identified the time frames with the strongest display of emotion, and rated the emotional intensity of each video on a 5-point Likert scale. After the initial stage of selection, two other researchers performed an emotion recognition task by watching the videos without audio to ensure that the emotions can be elicited without understanding the language. This data was used to refine the final selection of 20 professional videos and 20 amateur videos. In the video validation phase, 30 participants were asked to identify and rate the intensity of emotion felt. This article includes a description of the video selection method, a detailed list of the videos selected, and participants' responses and ratings of emotional intensity for the 40 videos.

## Specifications Table

**Table:** 

Subject	Experimental and Cognitive Psychology
Specific subject area	Selection and validation of emotional videos
Type of data	Table
How data were acquired	Professional videos were movie trailers from IMDb, an online database of information related to films, and amateur videos were from YouTube, an online video-sharing platform. Video validation data was collected online using Psychopy 3.0 and Pavlovia.
Data format	The videos were compressed in MP4 format. Video validation data was collected in Excel (.xlsx) format.
Parameters for data collection	To select professional videos, Western movie trailers were chosen from IMDb's Best Picture-Winning or Top Box Office by identifying scenes that display the common emotional triggers defined by Paul Ekman Group [8], [9], [10], [11], [12]. For amateur videos, YouTube videos were selected by searching for keywords of common emotional triggers [8], [9], [10], [11], [12]. For video validation, UMS students who are Chinese from China, Chinese from Malaysia, or Bumiputera from Malaysia were recruited.
Description of data collection	Based on the above criteria, researchers selected professional and amateur videos that displayed the five basic emotions and rated the emotional intensity of each video on a 5-point Likert scale. All videos were cut to less than 60s and were presented on Psychopy 3.0 and uploaded to Pavlovia. To refine the video selection, two other researchers performed the emotion recognition task by watching the videos without audio. This data is used to select the final 40 videos. To validate the videos, 30 participants were asked to identify and rate the emotional intensity of the videos.
Data source location	Institution: Universiti Malaysia Sabah City/Town/Region: Kota Kinabalu Country: Malaysia
Data accessibility	Repository name: Mendeley Data Direct URL to data: https://data.mendeley.com/datasets/fshjfvg4bt/draft?a=934d9407-548a-4e28-9331-26419429674c

## Value of the Data

• This dataset provides researchers with a collection of validated videos (i.e., professionally filmed movie trailers and amateur YouTube videos) and provides insight as to whether differences in cultural diversity (e.g., in China and Malaysia) affect emotion recognition.
• This dataset will benefit a range of researchers who investigate emotional or social skills, which includes cognitive psychologists, experimental psychologists, or social psychologists.
• The validated videos can be used in emotion-related experiments to understand various factors that affect emotion perception (e.g., facial expressions or body movement, methods of filming, audio or visual information, etc.).
• Based on the findings of future research, training programs or apps can be developed to teach and improve emotional and social skills in different settings and populations (e.g., in early education or with individuals with autism).

## 1 Data Description

The professional videos were selected from IMDb by identifying scenes that displayed the common emotional triggers defined by Paul Ekman Group [8], [9], [10], [11], [12]. The selection method, inclusion and exclusion criteria for professional videos are detailed in Table 1.

**Table 1:** Selection method, inclusion and exclusion criteria for professional videos.

Emotion	Selection Method	Inclusion Criteria	Exclusion Criteria
Happiness	IMDb – Best-picture winning (sorted by year descending) IMDb – Top box office (genre: comedy; title type: feature film; sorted by popularity)	Western movie trailers	Genre: Fantasy, historical fiction, animation Trailers filmed in black and white
Fear and Disgust	IMDb – Top box office (genre: horror; title type: feature film; sorted by popularity)		Genre: TV series, video games Trailers filmed in black and white
Anger and Sadness	IMDb – Best picture-winning (sorted by year descending)		Genre: Fantasy, historical fiction Trailers filmed in black and white

Table 2 includes a detailed list of the 40 professional and amateur videos that were presented in the video validation phase, including the movie names, video links and time frames, the common emotional triggers that are displayed in the videos [8], [9], [10], [11], [12], brief descriptions of the video content, the length of the videos, and the emotional intensity of the videos rated by the researchers.

**Table 2:** A detailed list of professional and amateur videos.

Emotion	Alias	Movie Name	Video Link (Time Frame)	Common Triggers [8], [9], [10], [11], [12]	Content Description	Length (sec)	Emotional Intensity
Happiness	PH1	Forrest Gump	https://www.imdb.com/title/tt0109830/?ref_=fn_al_tt_1 (2:07-2:25 + 3:27-3:42)	Experiencing something beautiful, surprising or amazing; feeling connected	A couple reuniting and envisioning a future together.	32	3.5
	PH2	Playing with Fire	https://www.imdb.com/title/tt9134216/?ref_=fn_al_tt_1 (1:40-2:01)	Witnessing or participating in acts of human goodness, kindness, and compassion; witnessing something humorous	Chaos while taking care of three children.	21	4
	PH3	Slumdog Millionaire	https://www.imdb.com/title/tt1010048/?ref_=fn_al_tt_1 (0:50-1:06 + 1:48-1:57)	Experiencing something beautiful, surprising or amazing; feeling connected	Recalling fond memories with loved ones.	25	4
	PH4	Home Alone 2: Lost in New York	https://www.imdb.com/title/tt0104431/?ref_=fn_al_tt_1 (0:05-0:47)	Witnessing something humorous or amusing	Burglars falling into traps that a child set.	42	3.5
	AH1	-	https://youtu.be/bF4xCJbJaho	Witnessing something humorous or amusing	Two pandas were playing together, and a sudden sneeze made people laugh.	29	5
	AH2	-	https://youtu.be/EulNYSsDQg8	Enjoyment derived through sound	A teacher pronounces "Google" in a different way and students laugh together.	13	3
	AH3	-	https://youtu.be/ewsO1tXbt5Y	Experiencing something beautiful, surprising or amazing	A little girl's father, who is in the military, surprises her at her birthday party.	59	5
	AH4	-	https://youtu.be/GbPsBmBpgT4	Witnessing or participating in acts of human goodness, kindness, and compassion	A little girl pretends to be a veterinarian and bandages a dog.	35	4
Fear	PF1	Countdown	https://www.imdb.com/title/tt10039344/?ref_=fn_al_tt_1 (1:45-2:14)	Darkness; death and dying	Running away from death and being captured by a ghostly hand.	29	4
	PF2	Doctor Sleep	https://www.imdb.com/title/tt5606664/?ref_=fn_al_tt_1 (0:37-0:44 + 2:08-2:16)	Darkness; death and dying	Being grabbed by a hand; being suffocated by hands.	15	4
	PF3	It	https://www.imdb.com/title/tt1396484/?ref_=nv_sr_srsg_8 (1:56-2:17)	Darkness; death and dying	A hand reaches out to grab children; clown appears unexpectedly and reaches out to grab a child.	21	4
	PF4	Scary Stories to Tell in the Dark	https://www.imdb.com/title/tt3387520/?ref_=nv_sr_srsg_0 (0:08-0:25)	Darkness; death and dying	Ghostly figure abruptly flies towards the screen.	18	4
	AF1	-	https://youtu.be/87uttrluFLI	Snakes	A person tries to capture a large snake that is hiding under a bed.	37	5
	AF2	-	https://youtu.be/kZZdGtoFYvk	Heights	A person hanging off a tall building with one hand; a person sliding off a tall, slanted building.	27	4
	AF3	-	https://youtu.be/rRBfi7YdQTk	Loss of visibility of surroundings	A big truck driver turns around on a narrow road.	36	3
	AF4	-	https://youtu.be/jfQ3F-6CydQ	Other animals	A performer teases a crocodile but got bitten by it.	28	3
Disgust	PD1	Jennifer's Body	https://www.imdb.com/title/tt1131734/?ref_=fn_al_tt_1 (0:57-1:02)	Perceived perversions or actions of other people	A girl burns her tongue with a lighter.	5	3
	PD2	Midsommar	https://www.imdb.com/title/tt8772262/?ref_=fn_al_tt_1 (2:10-2:15)	Expelled bodily products; something rotting, or dying; being exposed to bodily insides	Scenes of disfigured faces and bodies being dissected.	5	4
	PD3	The Shining	https://www.imdb.com/video/vi1476002073?ref_=tt_pv_vi_aiv_2 (1:00-1:12)	Expelled bodily products	Blood gushing out from the elevators and overflowing the room.	12	3
	PD4	The Babysitter	https://www.imdb.com/title/tt4225622/?ref_=adv_li_i (0:39-0:49)	Expelled bodily products; something rotting or dying	Someone was stabbed and blood is gushing out.	10	3
	AD1	-	https://youtu.be/M5_FZGryjyM	Being exposed to bodily insides	Surgical extraction of tooth.	20	4
	AD2	-	https://youtu.be/QxxNjtyh3RU	Certain foods (often from cultures other than our own)	A man eating an octopus and biting off its arms.	34	4
	AD3	-	https://youtu.be/dw3svc_-Pss	Expelled bodily products (vomit)	A person vomiting in bed.	26	5
	AD4	-	https://youtu.be/oOkRkF_Zi0k	Something rotting	An exposed wound was infected with maggots.	11	5
Anger	PA1	Argo	https://www.imdb.com/title/tt1024648/?ref_=vp_vi_tt (0:09-0:28)	Interference; injustice; betrayal, abandonment, rejection	Riot; hostages taken and held in gunshot.	19	3
	PA2	Chicago	https://www.imdb.com/title/tt0299658/?ref_=adv_li_i (0:56-1:01)	Someone trying to hurt us; another person's anger	Argument between two people, resulting in one person pushing another against the wall.	5	3.5
	PA3	12 Years of Slave	https://www.imdb.com/title/tt2024544/?ref_=fn_al_tt_1 (0:35-1:15)	Interference; injustice; someone trying to hurt us or a loved one; betrayal, abandonment, rejection	A man captured into slavery and being mistreated.	41	4
	PA4	The King's Speech	https://www.imdb.com/title/tt1504320/?ref_=fn_al_tt_2 (0:35-0:44 + 1:18-1:30)	Interference; another person's anger	King frustrated during preparation of speech.	21	3.5
	AA1	-	https://youtu.be/qAbp1Vn6s4M	Injustice	Father abuses a child.	37	4
	AA2	-	https://youtu.be/jwa4mkjdm7w	Interference	A passenger demands the bus driver to apologize by yelling at him.	21	4
	AA3	-	https://youtu.be/476G_F6K_MM	Hurt loved one physically	Husband abuses and hits his wife.	37	5
	AA4	-	https://youtu.be/ngnsgJzS58M	Betrayal	A woman repeatedly slaps an elderly man.	45	4
Sadness	PS1	Titanic	https://www.imdb.com/title/tt0120338/?ref_=fn_al_tt_1 (1:27-1:57)	Endings and goodbyes	Titanic is sinking and people are struggling to survive.	30	3.5
	PS2	Moonlight	https://www.imdb.com/title/tt4975722/?ref_=fn_al_tt_1 (0:36-1:17)	Rejection by a friend or lover; the loss of some aspect of identity	The struggles of being bullied by others, being yelled at, and being beaten.	42	4
	PS3	Crash	https://www.imdb.com/title/tt0375679/?ref_=fn_al_tt_1 (47-1:04 + 1:38-2:12)	Rejection by a friend or lover; the loss of some aspect of identity; sickness or death of a loved one	Brother being locked away for stealing car; car crashes.	41	4
	PS4	Unforgiven	https://www.imdb.com/title/tt0105695/?ref_=fn_al_tt_1 (1:03-1:40)	Endings and goodbyes; death of a loved one	A man is shaving while overlooking a grave. He brings flowers to the grave and sits there in silence.	37	3
	AS1	-	https://youtu.be/GNr8mekkzZA	Death of a loved one	An elder brother crying while looking at the photo of his younger brother who passed away.	52	3
	AS2	-	https://youtu.be/aiC6jChe6-U	Being disappointed by an unexpected outcome	A boy sharing about his friend who passed away unexpectedly.	22	4
	AS3	-	https://youtu.be/n6MoHWQvqJo	Endings and goodbyes	A woman has to part with her dog and cries while hugging her dog.	20	4
	AS4	-	https://youtu.be/7caJWZdS0UA	Sickness of a loved one	A dog hears continuous barking but couldn't move. The dog cries helplessly.	33	5

In the video validation phase, three groups of participants watched the videos, identified the emotion felt, and rated the emotional intensity of each video. The data is reported in Table 3. The raw data are available in the Mendeley dataset.

**Table 3:** The count and percentage of emotion detected and the mean of emotional intensity.

			Response Count (Percentage)				
Emotion	Alias	Emotion Detected	Chinese from China	Chinese from Malaysia	Bumiputera from Malaysia	Total Count (Total Percentage)	Mean of Emotional Intensity
Happiness	PH1	Happiness	10 (100%)	9 (90%)	7 (70%)	26 (86.67%)	3.50
		Sadness	0 (0%)	1 (10%)	3 (30%)	4 (13.33%)	2.50
	PH2*	Happiness	10 (100%)	10 (100%)	10 (100%)	30 (100%)	3.40
	PH3	Happiness	8 (80%)	9 (90%)	8 (80%)	25 (83.33%)	3.00
		Fear	0 (0%)	0 (0%)	1 (10%)	1 (3.33%)	1.00
		Disgust	0 (0%)	0 (0%)	1 (10%)	1 (3.33%)	3.00
		Sadness	2 (20%)	1 (10%)	0 (0%)	3 (10%)	2.67
	PH4*	Happiness	10 (100%)	8 (80%)	8 (80%)	26 (86.67%)	3.38
		Fear	0 (0%)	1 (10%)	1 (10%)	2 (6.67%)	1.50
		Disgust	0 (0%)	1 (10%)	0 (0%)	1 (3.33%)	3.00
		Sadness	0 (0%)	0 (0%)	1 (10%)	1 (3.33%)	1.00
	AH1	Happiness	10 (100%)	10 (100%)	8 (80%)	28 (93.33%)	3.71
		Sadness	0 (0%)	0 (0%)	2 (20%)	2 (6.67%)	3.00
	AH2*	Happiness	10 (100%)	9 (90%)	10 (100%)	29 (96.67%)	3.59
		Fear	0 (0%)	1 (10%)	0 (0%)	1 (3.33%)	3.00
	AH3	Happiness	10 (100%)	8 (80%)	10 (100%)	28 (93.33%)	4.11
		Sadness	0 (0%)	2 (20%)	0 (0%)	2 (6.67%)	4.00
	AH4*	Happiness	10 (100%)	10 (100%)	9 (90%)	29 (96.67%)	3.52
		Sadness	0 (0%)	0 (0%)	1 (10%)	1 (3.33%)	2.00
Fear	PF1*	Fear	10 (100%)	9 (90%)	10 (100%)	29 (96.67%)	3.97
		Disgust	0 (0%)	1 (10%)	0 (0%)	1 (3.33%)	5.00
	PF2*	Fear	10 (100%)	10 (100%)	10 (100%)	30 (100%)	3.33
	PF3	Fear	10 (100%)	9 (90%)	10 (100%)	29 (96.67%)	3.93
		Disgust	0 (0%)	1 (10%)	0 (0%)	1 (3.33%)	3.00
	PF4	Fear	10 (100%)	10 (100%)	9 (90%)	29 (96.67%)	3.34
		Disgust	0 (0%)	0 (0%)	1 (10%)	1 (3.33%)	3.00
	AF1	Happiness	0 (0%)	0 (0%)	1 (10%)	1 (3.33%)	2.00
		Fear	9 (90%)	9 (90%)	5 (50%)	23 (76.67%)	3.30
		Disgust	1 (10%)	0 (0%)	4 (40%)	5 (16.67%)	3.60
		Sadness	0 (0%)	1 (10%)	5 (50%)	1 (3.33%)	3.00
	AF2*	Happiness	0 (0%)	3 (30%)	2 (20%)	5 (16.67%)	3.00
		Fear	10 (100%)	7 (70%)	8 (80%)	25 (83.33%)	3.64
	AF3*	Fear	10 (100%)	10 (100%)	10 (100%)	30 (100%)	3.43
	AF4	Happiness	0 (0%)	0 (0%)	1 (10%)	1 (3.33%)	4.00
		Fear	10 (100%)	6 (60%)	7 (70%)	23 (76.67%)	3.83
		Disgust	0 (0%)	2 (20%)	2 (20%)	4 (13.33%)	4.75
		Sadness	0 (0%)	2 (20%)	0 (0%)	2 (6.67%)	2.50
Disgust	PD1*	Happiness	0 (0%)	0 (0%)	1 (10%)	1 (3.33%)	1.00
		Fear	2 (20%)	3 (30%)	2 (20%)	7 (23.33%)	2.71
		Disgust	8 (80%)	6 (60%)	7 (70%)	21 (70%)	3.10
		Sadness	0 (0%)	1 (10%)	0 (0%)	1 (3.33%)	2.00
	PD2*	Fear	3 (30%)	1 (10%)	3 (30%)	7 (23.33%)	4.14
		Disgust	7 (70%)	6 (60%)	7 (70%)	20 (66.67%)	3.35
		Anger	0 (0%)	1 (10%)	0 (0%)	1 (3.33%)	3.00
		Sadness	0 (0%)	2 (20%)	0 (0%)	2 (6.67%)	2.50
	PD3	Fear	3 (30%)	4 (40%)	4 (40%)	11 (36.67%)	3.73
		Disgust	7 (70%)	5 (50%)	6 (60%)	18 (60%)	3.06
		Sadness	0 (0%)	1 (10%)	0 (0%)	1 (3.33%)	2.00
	PD4	Happiness	0 (0%)	0 (0%)	3 (30%)	3 (10%)	2.67
		Fear	3 (30%)	0 (0%)	3 (30%)	6 (20%)	4.00
		Disgust	6 (60%)	8 (80%)	4 (40%)	18 (60%)	2.89
		Anger	0 (0%)	1 (10%)	0 (0%)	1 (3.33%)	4.00
		Sadness	1 (10%)	1 (10%)	0 (0%)	2 (6.67%)	3.50
	AD1	Fear	1 (10%)	0 (0%)	1 (10%)	2 (6.67%)	3.00
		Disgust	9 (90%)	9 (90%)	9 (90%)	27 (90%)	4.00
		Sadness	0 (0%)	1 (10%)	0 (0%)	1 (3.33%)	2.00
	AD2	Happiness	0 (0%)	1 (10%)	1 (10%)	2 (6.67%)	3.00
		Fear	0 (0%)	0 (0%)	1 (10%)	1 (3.33%)	3.00
		Disgust	10 (100%)	9 (90%)	8 (80%)	27 (90%)	3.89
	AD3*	Happiness	0 (0%)	0 (0%)	1 (10%)	1 (3.33%)	4.00
		Disgust	10 (100%)	10 (100%)	8 (80%)	28 (93.33%)	4.04
		Sadness	0 (0%)	0 (0%)	1 (10%)	1 (3.33%)	1.00
	AD4*	Fear	0 (0%)	2 (20%)	0 (0%)	2 (6.67%)	4.50
		Disgust	10 (100%)	7 (70%)	10 (100%)	27 (90%)	4.37
		Sadness	0 (0%)	1 (10%)	0 (0%)	1 (3.33%)	2.00
Anger	PA1	Happiness	0 (0%)	0 (0%)	1 (10%)	1 (3.33%)	1.00
		Fear	3 (30%)	4 (40%)	4 (40%)	11 (36.67%)	2.82
		Disgust	0 (0%)	1 (10%)	2 (20%)	3 (10%)	3.00
		Anger	6 (60%)	4 (40%)	2 (20%)	12 (40%)	3.58
		Sadness	1 (10%)	1 (10%)	1 (10%)	3 (10%)	4.00
	PA2	Happiness	0 (0%)	0 (0%)	3 (30%)	3 (10%)	4.00
		Fear	2 (20%)	4 (40%)	0 (0%)	6 (20%)	3.67
		Disgust	0 (0%)	0 (0%)	2 (20%)	2 (6.67%)	3.00
		Anger	7 (70%)	5 (50%)	5 (50%)	17 (56.67%)	3.71
		Sadness	1 (10%)	1 (10%)	0 (0%)	2 (6.67%)	2.00
	PA3*	Fear	0 (0%)	0 (0%)	1 (10%)	1 (3.33%)	1.00
		Disgust	0 (0%)	0 (0%)	1 (10%)	1 (3.33%)	1.00
		Anger	8 (80%)	7 (70%)	8 (80%)	23 (76.67%)	2.52
		Sadness	2 (20%)	3 (30%)	0 (0%)	5 (16.67%)	3.00
	PA4*	Happiness	0 (0%)	1 (10%)	3 (30%)	4 (13.33%)	3.50
		Fear	0 (0%)	1 (10%)	1 (10%)	2 (6.67%)	3.50
		Disgust	1 (10%)	0 (0%)	0 (0%)	1 (3.33%)	2.00
		Anger	7 (70%)	6 (60%)	5 (50%)	18 (60%)	3.33
		Sadness	2 (20%)	2 (20%)	1 (10%)	5 (16.67%)	2.60
	AA1	Fear	0 (0%)	0 (0%)	1 (10%)	1 (3.33%)	4.00
		Disgust	1 (10%)	1 (10%)	1 (10%)	3 (10%)	4.67
		Anger	9 (90%)	7 (70%)	8 (80%)	24 (80%)	4.63
		Sadness	0 (0%)	2 (20%)	0 (0%)	2 (6.67%)	3.00
	AA2*	Happiness	0 (0%)	1 (10%)	0 (0%)	1 (3.33%)	3.00
		Disgust	0 (0%)	1 (10%)	0 (0%)	1 (3.33%)	3.00
		Anger	10 (100%)	8 (80%)	8 (80%)	26 (86.67%)	3.73
		Sadness	0 (0%)	0 (0%)	2 (20%)	2 (6.67%)	2.50
	AA3*	Disgust	0 (0%)	0 (0%)	1 (10%)	1 (3.33%)	5.00
		Anger	10 (100%)	8 (80%)	9 (90%)	27 (90%)	4.44
		Sadness	0 (0%)	2 (20%)	0 (0%)	2 (6.67%)	3.00
	AA4	Fear	1 (10%)	0 (0%)	1 (10%)	2 (6.67%)	3.00
		Disgust	0 (0%)	0 (0%)	1 (10%)	1 (3.33%)	4.00
		Anger	9 (90%)	7 (70%)	7 (70%)	23 (76.67%)	4.52
		Sadness	0 (0%)	3 (30%)	1 (10%)	4 (13.33%)	3.25
Sadness	PS1	Happiness	1 (10%)	1 (10%)	1 (10%)	3 (10%)	2.33
		Fear	1 (10%)	2 (20%)	1 (10%)	4 (13.33%)	3.00
		Sadness	8 (80%)	7 (70%)	8 (80%)	23 (76.67%)	3.26
	PS2	Fear	1 (10%)	1 (10%)	1 (10%)	3 (10%)	3.67
		Anger	3 (30%)	0 (0%)	3 (30%)	6 (20%)	3.67
		Sadness	6 (60%)	9 (90%)	6 (60%)	21 (70%)	3.00
	PS3*	Happiness	0 (0%)	1 (10%)	1 (10%)	2 (6.67%)	3.50
		Sadness	10 (100%)	9 (90%)	9 (90%)	28 (93.33%)	3.61
	PS4*	Happiness	1 (10%)	0 (0%)	0 (0%)	1 (3.33%)	1.00
		Fear	0 (0%)	1 (10%)	1 (10%)	2 (6.67%)	1.50
		Sadness	9 (90%)	9 (90%)	9 (90%)	27 (90%)	3.19
	AS1*	Sadness	10 (100%)	10 (100%)	10 (100%)	30 (100%)	3.87
	AS2	Happiness	0 (0%)	1 (10%)	0 (0%)	1 (3.33%)	3.00
		Anger	0 (0%)	0 (0%)	1 (10%)	1 (3.33%)	4.00
		Sadness	10 (100%)	9 (90%)	9 (90%)	28 (93.33%)	3.54
	AS3*	Disgust	1 (10%)	0 (0%)	0 (0%)	1 (3.33%)	4.00
		Sadness	9 (90%)	10 (100%)	10 (100%)	29 (96.67%)	3.66
	AS4	Fear	2 (20%)	0 (0%)	0 (0%)	2 (6.67%)	2.50
		Sadness	8 (80%)	10 (100%)	10 (100%)	28 (93.33%)	3.71

⁎ Videos with the two highest recognition accuracy in each category were selected. If the recognition accuracy was the same for multiple videos, the video with the least confusion with another emotion and/or the video with the highest mean of emotional intensity was selected.

## 2 Experimental Design, Materials and Methods

Emotion recognition is an important social skill that allows individuals to understand others’ mental state and to respond appropriately in different social situations [1], [2], [3]. Research utilized different types of stimuli to investigate emotion perception, including computer-generated emoticons, photos of posed facial expressions, and videos that more closely represent social interactions [4], [5], [6], [7]. To create a dataset of professional and amateur videos that can be used in emotion perception research, video selection and video validation phases were conducted to identify emotional videos that elicit five basic emotions (i.e., happiness, fear, disgust, anger, and sadness). In the video selection phase, researchers selected emotional videos that displayed the common emotional triggers defined by Paul Ekman Group [8], [9], [10], [11], [12], identified the time frames with the strongest display of emotion, and rated the emotional intensity of each video on a 5-point Likert scale.

For professional videos, Western movie trailers were selected from IMDb, which is an online database of information related to films, by identifying scenes that displayed the common emotional triggers [8], [9], [10], [11], [12]. All videos were chosen from IMDb's Best Picture-Winning or Top Box Office. Additionally, inclusion and exclusion criteria were set to ensure that the filming style and genre of all videos are similar (see details of selection method, inclusion and exclusion criteria in Table 1). For amateur videos, videos were selected from YouTube, which is an online video-sharing platform, by searching for keywords of common emotional triggers [8], [9], [10], [11], [12], [13], [14]. The selected videos were trimmed to less than 60 seconds using the Quick Editing software. Format Factory, which is a multimedia production software, was used to change the format of the videos to MP4. The resolution of the videos is 720 × 480 and the frame rate is 29.97fps.

After the initial stage of video selection, two researchers performed the emotion recognition task, which was presented on the Psychopy 3.0 software and uploaded to Pavlovia. The videos were played without audio to ensure that the emotions can be elicited without understanding the language. Upon completion, this data was used to refine the final selection of 20 professional videos and 20 amateur videos (see Table 2).

In the video validation phase, 30 undergraduate and postgraduate students (14 males and 16 females) enrolled in Universiti Malaysia Sabah were recruited. 10 participants were Chinese from China (6 males and 4 females, mean age 21.1 years, ranging from 19 to 23, SD = 1.197), 10 participants were Chinese from Malaysia (5 males and 5 females, mean age 20.5 years, ranging from 19 to 26, SD = 2.461), and 10 participants were Bumiputera from Malaysia (3 males and 7 females, mean age 20.9 years, ranging from 18 to 23, SD = 1.792). All participants, who had normal or corrected vision, were recruited through convenience sampling and snowball sampling. All participants gave informed consent prior to their participation.

The video validation phase was carried out using the Psychopy 3.0 software, which was uploaded to Pavlovia. All participants accessed the videos via a link that was provided by the researcher and performed the emotion recognition task on their respective laptops. The 40 videos were presented randomly, and each video was followed by three questions (i.e., What emotion did you feel after watching the video? How strongly did you feel the emotion? Have you ever watched this video before?). Participants who have watched more than 60% of the videos prior to the video validation phase were excluded from the data as they may have anticipated the content of the videos [14].

Upon completion, the data on the count and percentage of emotion detected and the rating of emotional intensity for each video were recorded on Pavlovia and then transferred to XLSX worksheet (.xlsx) to calculate the total count and total percentage of detected emotions and the mean of emotional intensity (see Table 3). In Table 3, 20 videos (indicated with asterisks) can be used for future emotion-related research. Videos with the two highest recognition accuracy in each category were selected. The recognition accuracy of all videos was higher than 60%. If the recognition accuracy was the same for multiple videos, the video with the least confusion with another emotion and/or the video with the highest mean of emotional intensity was selected.

## Ethics Statement

Ethics was approved by the ethical committee of the Faculty of Medicine and Health Sciences, Universiti Malaysia Sabah [approval code: JKEtika 1/19 (20)]. Informed consent was obtained from all participants.

## Declaration of Competing Interest

The authors declare that there is no conflict of interest.

## References

[1] Matsumoto D., Hwang H.S. (2013). Facial expressions. Nonverbal Communication Science Application.

[2] Krumhuber E.G., Skora L., Müller B., Wolf S.I. (2016). Perceptual Study on Facial Expressions. Handbook of Human Motion.

[3] Gross A.L., Ballif B. (1991). Children's understanding of emotion from facial expressions and situations: a review. Dev. Rev..

[4] Yuki M., Maddux W.W., Masuda T. (2007). Are the windows to the soul the same in the East and West? Cultural differences in using the eyes and mouth as cues to recognize emotions in Japan and the United States. J. Exp. Soc. Psychol..

[5] Tan C.B.Y., Sheppard E., Stephen I.D. (2015). A change in strategy: Static emotion recognition in Malaysian Chinese. Cogent Psychol.

[6] Rumpa L.D., Wibawa A.D., Purnomo M.H., Tulak H. (2016). Validating video stimulus for eliciting human emotion: A preliminary study for e-health monitoring system. Proceedings of the 4th International Conference on Instrumentation, Communication Information Technology and Biomedical Engineering ICICI-BME 2015.

[7] Baveye Y., Dellandréa E., Chamaret C., Chen L. (2015). LIRIS-ACCEDE: a video database for affective content analysis. IEEE Trans. Affect. Comput..

[8] Paul Ekman Group, Enjoyment, 2020, Paul Ekman Group LLC https://www.paulekman.com/universal-emotions/what-is-enjoyment/, Accessed 13 March 2020.

[9] Paul Ekman Group, Fear, 2020, Paul Ekman Group LLC https://www.paulekman.com/universal-emotions/what-is-fear/, Accessed 13 March 2020.

[10] Paul Ekman Group, Disgust, 2020, Paul Ekman Group LLC https://www.paulekman.com/universal-emotions/what-is-disgust/, Accessed 13 March 2020.

[11] Paul Ekman Group, Anger, 2020, Paul Ekman Group LLC https://www.paulekman.com/universal-emotions/what-is-anger/, Accessed 13 March 2020.

[12] Paul Ekman Group, Sadness, 2020, Paul Ekman Group LLC. https://www.paulekman.com/universal-emotions/what-is-sadness/, Accessed 13 March 2020.

[13] Quigley K.S., Lindquist K.A., Barrett L.F. (2014). Inducing and measuring emotion and affect. Handb. Res. Methods Soc. Personal. Psychol..

[14] Philippot P. (1993). Inducing and assessing differentiated emotion-feeling states in the laboratory. Cogn.Emot.

